# Incidental Axillary Dose of Tomotherapy in Hypofractionated Whole Breast Radiotherapy for Early Breast Cancer: A Dosimetrical Analysis

**DOI:** 10.3390/medicina59061081

**Published:** 2023-06-03

**Authors:** Seung-Gu Yeo, Cheol Wan Lim, Sung-Mo Hur, Zisun Kim, Kwang Hwan Cho, Min-Jeong Kim

**Affiliations:** 1 Department of Radiation Oncology, Soonchunhyang University College of Medicine, Soonchunhyang University Hospital, Bucheon 14584, Republic of Korea; 2 Department of Surgery, Soonchunhyang University College of Medicine, Soonchunhyang University Hospital, Bucheon 14584, Republic of Korea; 3 Department of Radiology, Hallym University College of Medicine, Hallym University Sacred Heart Hospital, Anyang 14068, Republic of Korea

**Keywords:** breast cancer, radiation therapy, tomotherapy, incidental dose, axilla

## Abstract

*Background and Objectives*: Intensity-modulated radiation therapy (IMRT) is becoming a more common method of performing whole breast irradiation (WBI) for early breast cancer. This study aimed to examine the incidental dose to the axillary region using tomotherapy, a unique form of IMRT. *Patients and Methods*: This study included 30 patients with early-stage breast cancer who underwent adjuvant WBI using TomoDirect IMRT. A hypofractionation scheme of 42.4 Gy delivered in 16 fractions was prescribed. The plan comprised of two parallel-opposed beams, along with two additional beams positioned anteriorly at gantry angles of 20° and 40° from the medial beam. The incidental dose received at axillary levels I, II, and III was evaluated using several dose-volume parameters. *Results*: The study participants had a median age of 51 years, and 60% had left-sided breast cancer. The mean dose of the axilla for levels I, II, and III were 15.5 ± 4.8 Gy, 14.9 ± 4.2 Gy, and 1.5 ± 1.6 Gy, respectively. Adequate coverage of the axilla, defined as V95%[%], was achieved for 4.7 ± 3.9%, 4.8 ± 3.7%, and 0 ± 0% for levels I, II, and III, respectively. The results were compared with those of previously published studies, and the axillary mean dose and V95%[%] of TomoDirect IMRT were low, comparable to other IMRT techniques, and lower than those of traditional tangential therapy. *Conclusions*: While incidental axillary radiation during WBI has been proposed to assist in regional disease control, the TomoDirect plan was demonstrated to decrease this dose, and a hypofractionation scheme would further lower its biological effectiveness. Future clinical studies should incorporate dosimetrical analysis of incidental axillary dose, in order to facilitate hypofractionated IMRT planning with risk-adjusted axilla coverage in early breast cancer.

## 1. Introduction

Whole breast radiation therapy (RT) has long been performed for early-stage breast cancer using two- or three-dimensional parallel-opposed tangential fields. However, this method inevitably spreads some portion of the prescribed dose to the ipsilateral axilla, which is known as the incidental axillary dose [1]. It has been suggested that this unintended dose contributes to the eradication of occult microscopic disease in the undissected axilla [2]. To replace this less advanced RT technique, intensity-modulated RT (IMRT) is now increasingly being used for RT in breast cancer [3]. In IMRT for whole breast irradiation (WBI) of early breast cancer, the axilla is usually not considered a target volume or organ at risk (OAR) during plan creation. Nonetheless, several dosimetrical studies have demonstrated that the incidental axillary dose is lower in IMRT than in traditional tangential treatment [1]. This is due to the fact that IMRT is an advanced conformal RT technique characterized by a steep dose gradient and better sparing of adjacent normal tissues. 

Tomotherapy (Accuray Inc., Sunnyvale, CA, USA) is an RT platform capable of delivering highly conformal IMRT plans within a helical geometry under image guidance. Compared to conventional IMRT, tomotherapy’s unique design features improve the dose conformity and homogeneity of target volumes, while also providing conformal avoidance of adjacent normal organs [4]. In cases of WBI without regional nodal irradiation (RNI), TomoDirect is considered a more suitable option than helical tomotherapy. TomoDirect utilizes static gantry positions combined with simultaneous couch translation and dynamic collimator modulation. This approach avoids the low-dose integral spray to the lung and long treatment times associated with helical methods by confining dose delivery to a few specific angles [5]. By using such sophisticated conformal irradiation techniques, the incidental dose to axillary lymph drainage routes can be further reduced.

To date, only one study has analyzed the incidental axillary dose using tomotherapy, which was based on helical tomotherapy, not TomoDirect [6]. In addition, the results of this study appear to provide inadequate information due to critical limitations in the study methods. 

The purpose of the present study is to conduct a dosimetrical analysis of the incidental dose of the axillary region in patients with early-stage breast cancer who underwent adjuvant hypofractionated WBI using TomoDirect.

## 2. Materials and Methods

This study included 30 patients with early breast cancer who underwent breast-conserving surgery and adjuvant hypofractionated WBI using TomoDirect in 2022. Patients who were treated with neoadjuvant chemotherapy, any RNI as part of the target volume, or prior breast augmentation were excluded. The clinical and pathological characteristics of the patients are presented in Table 1. 

For simulation, patients were immobilized on a breast-tilting board with both arms abducted above the head. Axial computed tomography slices were obtained at 3 mm intervals using SOMATOM Confidence (Siemens Healthcare, Erlangen, Germany) and were transferred to MIM Maestro (MIM Software, Cleveland, OH, USA) for contouring the regions of interest. The clinical target volume (CTV) of the breast, and the ipsilateral axillary nodal levels I-III, were contoured according to the European Society for Radiotherapy and Oncology contouring guidelines [7]. The planning target volume (PTV) was created by expanding the CTV by 5 mm in all directions and then cropping 3 mm of skin from the surface of the body. The OAR included the ipsilateral and contralateral lungs, the heart, the contralateral breast, the spinal cord, and the ipsilateral humerus head.

A hypofractionation scheme of 42.4 Gy delivered in 16 fractions was prescribed, and the plan was normalized such that 95% of the PTV receives prescription dose. The dose-volume constraints for the OAR followed the NRG whole breast hypofractionation protocol, as detailed: ≤15% of the ipsilateral lung should receive ≥16 Gy, ≤20% of the ipsilateral lung should receive ≥8 Gy, ≤30% of the ipsilateral lung should receive ≥4 Gy, and ≤15% of the contralateral lung should receive ≥4 Gy. For left-sided breast cancers, ≤10% of the whole heart should receive ≥16 Gy and the mean heart dose should be ≤4.2 Gy [8]. WBI was planned using Accuracy Precision (version 3.3.1.3, Accuray Inc., Sunnyvale, CA, USA) with four gantry angles. Two tangential parallel-opposed beams were arranged to cover the PTV, minimize doses to the ipsilateral lung, and avoid irradiation to the contralateral breast. Two additional beams were generated by adjusting the gantry angles to 20° and 40°, respectively, anteriorly from the medial tangential beam. The field size was expanded for four binary multileaf collimator leaves (margin, 25.0 mm) on the outer edges to cover the flash region. TomoDirect IMRT plans with a field width of 5 cm in dynamic jaw mode were created. The plans were optimized using a modulation factor of 2.4 and a pitch of 0.5, with dose-volume histogram (DVH) points adjusted throughout the optimization to meet the OAR dose constraints and PTV coverage. The 6-MV photon beam was used for each field. The delineated axillary nodal region was not labeled as target or OAR and did not affect treatment planning. In general, a boost dose of four fractions was delivered via a separate plan.

The dose administered to the axillary lymph node levels during the whole breast treatment was analyzed, excluding the boost plan. A DVH was constructed for each plan. The incidental dose received at axillary levels I, II, and III was assessed based on several parameters, including the mean dose (Dmean), volume[%] receiving 95% of the prescribed dose (V95%[%]), V90%[%], V80%[%], and V50%[%]. Variation was expressed in standard deviations. Statistical analysis was carried out using SPSS software (version 26.0, SPSS Inc., Chicago, IL, USA).

## 3. Results

The patients’ median age was 51 years, with a range of 40 to 66 years. Of the patients, 26 (86.6%) had invasive ductal carcinoma and 2 (6.7%) had invasive lobular carcinoma. Eighteen (60%) had left-sided breast cancer. Most of the patients, i.e., 28 (93.3%), had negative sentinel lymph node, while 2 (6.7%) had micrometastatic disease in the sentinel lymph node. The clinical and pathological features of the patients are presented in Table 1.

**Figure 1:**
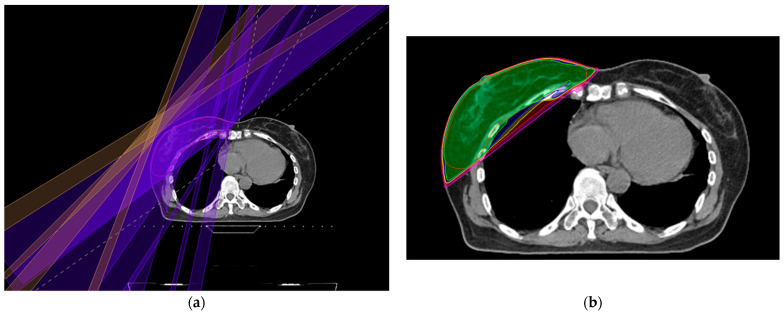
Representative TomoDirect IMRT plan: (**a**) four fields consisting of two tangential opposed beams plus two additional beams with gantry angles 20° and 40°, respectively, anterior from the medial tangential beam (red = planning target volume); (**b**) isodose distribution from the plan (**a**) (light green = 100% isodose line; blue = 95%; yellow = 90%; red = 80%, pink = 50%).

The average volume of the CTV for the breast was 486.8 ± 233.8 cm^3^, and the PTV had an average volume of 670.6 ± 267.8 cm^3^. Regarding the axillary nodal region, the mean volumes were 54.7 ± 18.5 cm^3^, 37.2 ± 8.5 cm^3^, and 9.0 ± 2.6 cm^3^ for levels I, II, and III, respectively. Figure 1 displays an axial image of the representative RT plan that shows the TomoDirect fields and the dose distributions.

**Figure 2:**
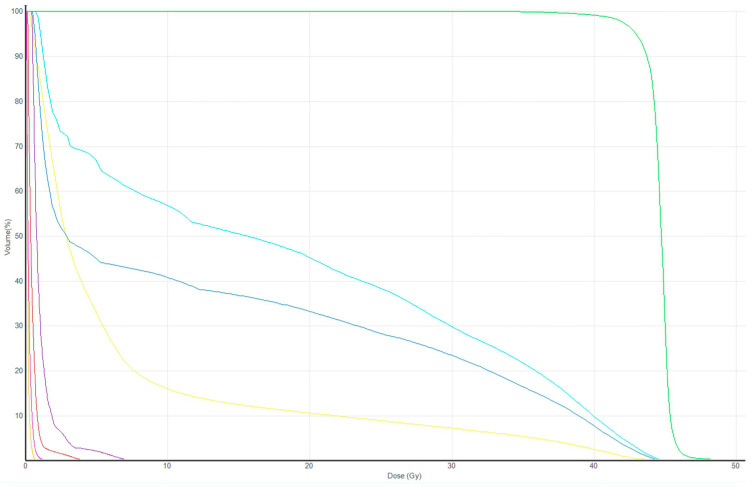
Dose-volume histogram of the Figure 1 plan: light blue = axilla level I; blue = axilla level II, purple = axilla level III; light green = planning target volume; yellow = right lung; orange = left lung; red = heart; pink = contralateral breast.

The Dmean values of the axilla for levels I, II, and III were 15.5 ± 4.8 Gy, 14.9 ± 4.2 Gy, and 1.5 ± 1.6 Gy, respectively. This Dmean of levels I, II, and III were 35.5%, 35.1%, and 3.5% of the prescribed dose, respectively. Adequate coverage of the axilla, defined as V95%[%], was achieved for 4.7 ± 3.9%, 4.8 ± 3.7%, and 0 ± 0% for levels I, II, and III, respectively. Further details regarding the dose-volume parameters of the axilla levels are shown in Table 2. Additionally, Figure 2 presents the DVH of plan in Figure 1.

Due to the lack of traditional tangential treatment or planning options at our facility, the results obtained from TomoDirect are compared with those of previously published studies. Table 3 presents the dose–volume data of representative studies on incidental axillary dose from WBI. As the prescription scheme in those studies was normo-fractionation (50 Gy/25 fractions), the percentage of prescription dose was primarily described in the table. The Dmean (%) and V95%[%] of all axilla levels of the present study were found to be lower than those achieved using standard tangents. The Dmean (%) and V95%[%] of axilla level I were between static IMRT and volumetric modulated arc therapy. The Dmean (%) and V95%[%] of axilla level II were slightly higher than static IMRT or volumetric modulated arc therapy. The Dmean (%) of axilla level III was lower than static IMRT or volumetric modulated arc therapy.

## 4. Discussion

Hitherto, there was only one tomotherapy study that has examined incidental axillary dose during WBI [6]. This study, conducted by Mayinger et al., evaluated 60 patients, with half receiving Helical tomotherapy and the other half receiving three-dimensional tangential therapy. The average doses in axillary lymph node levels I, II, and III were 31.6 Gy, 8.43 Gy, and 2.38 Gy for tomotherapy, and 24.0 Gy, 11.2 Gy, and 3.97 Gy for tangential therapy. The authors reported that the dose at axilla level I was significantly higher with tomotherapy compared to tangential therapy. However, it is crucial to be careful when interpreting this result. Patients receiving tomotherapy were administered a total dose of 50.4 Gy (1.8 Gy/fraction) along with a simultaneous integrated boost (2.25 Gy/fraction, total 63 Gy) to the surgical bed. On the other hand, patients receiving tangential therapy (50 Gy, 2.0 Gy/fraction) were given a sequential boost (10 or 16 Gy) that was not included in the analysis. Furthermore, 16 (53.3%) of the 30 patients in the Tomotherapy group had a tumor location at upper outer quadrant where treated with simultaneous integrated boost. This factor definitely contributed to the dose in axilla level I and was a critical limitation of the study. When the integrated boost dose in the tomotherapy was excluded, the dose at axilla level I may have been lower than that of tangential therapy. Additionally, tomotherapy was compared with tangential therapy in different patient groups. Breast shape and RT techniques both influence the amount of incidental axillary dose [12]. All other previous IMRT studies compared IMRT with tangential plans in the same patient group [11,13,14,15,16]. Although the V45Gy[%] in their research is not identical to the V95%[%] because of the simultaneous integrated boost, the average of 30.0% at level I was substantially higher than the 4.7% in our study. The variation in tomotherapy techniques (helical vs. direct), contouring guidelines, OAR constraints, and optimization parameters might also account for the variation in incidental axillary dose.

IMRT is increasingly being used as an adjuvant RT for breast cancer due to its dosimetrical benefits. These advantages include improved homogeneity of the breast target dose and a steeper dose gradient around the target volume, resulting in fewer radiation-related complications [3]. While the axilla is not considered an OAR in IMRT planning, several IMRT dosimetry studies have shown that the highly conformal dose distribution in IMRT delivers lower doses to the axilla than conventional tangential therapy [1]. Our findings are consistent with these previous IMRT studies (Table 2 and Table 3). This raises concerns about the potential risk of missing opportunities for regional control of occult axillary metastasis, particularly for patients with limited positive sentinel lymph nodes who do not undergo axillary lymph node dissection.

The ACOSOG Z0011 trial found that there were no significant differences in overall survival and local control for women with T1 or T2, cN0 breast cancer with 1-2 positive sentinel lymph nodes between those who underwent sentinel lymph node biopsy alone or completion axilla lymph node dissection [17]. Notably, the regional recurrence rate after sentinel lymph node biopsy alone was very low (0.9%), despite the fact that approximately 27% of these patients should have had additional lymph node metastases based on the results of axilla lymph node dissection group [18]. Both study arms received WBI using traditional tangential fields. However, in approximately 50% of the patients, high tangents were used, which should result in a higher axillary dose compared to standard tangents. Additionally, 17% of patients received RNI including supraclavicular fields [2]. Some experts have suggested that incidental axilla dose from tangential fields may play a role in axillary regional control [1]. While the prevailing consensus is that a radiation dose of approximately 45–50 Gy in 2 Gy per fraction is necessary to eradicate microscopic disease, the question of how much incidental axillary dose is adequate as prophylactic therapy for microscopically positive axilla remains unanswered [13]. Caution was advised when extrapolating the results of Z0011 to patients who receive adjuvant RT in which substantially less of the axilla is involved, such as partial breast irradiation [2]. It is conceivable that the utilization of sophisticated conformal IMRT techniques in WBI, such as the TomoDirect as in our study, may also fall into a similar category.

An additional point to consider is that the recommended standard for adjuvant WBI now involves a moderate hypofractionation scheme, which typically entails administering 40–42.5 Gy in 15–16 fractions [19]. In the present study, a hypofractionated dosage of 42.4 Gy in 16 fractions was used. Borm et al. investigated the impact of this fractionation change on the incidental dose to the axilla during WBI and found that, according to radiobiological models, the mean biological effective dose and tumor control probability in the axillary lymph nodes were significantly lower for hypofractionated schedules compared to conventional fractionation [20].

Therefore, hypofractionated IMRT, as shown in this study, has the possibility of not being able to achieve regional control of hidden axillary metastasis, especially in patients with limited positive sentinel lymph nodes who skip axillary lymph node dissection. The current National Comprehensive Cancer Network guidelines state that for patients with T1 or T2, cN0, and 1–2 positive sentinel lymph nodes who have not received preoperative chemotherapy, the use of comprehensive RNI with or without intentional inclusion of the axilla is at the discretion of the radiation oncologist [21]. In this situation, and when using hypofractionated IMRT, including axillary levels I-II (the typical levels removed during axillary lymph node dissection) in the target may be a reasonable alternative to using either WBI alone or WBI plus comprehensive RNI. The PORT-N1 trial is a multicenter, randomized clinical trial comparing the outcomes of control (WBI plus RNI) and experimental (WBI alone) groups for patients with pN1 (1–3 positive nodes) breast cancer [22]. The RNI in this trial has a broader definition, including irradiation only to axilla levels I and II in cases of high tangent field, based on the opinions of several experts who prefer a reduced regional radiation field for low risk pN1 breast cancer patients. IMRT, which includes axilla levels I-II in its target, provides more comprehensive coverage of axillary levels I-II compared to high tangents without increasing radiation exposure to the OAR, as expected from the features of IMRT [9]. Nonetheless, incorporating even a restricted RNI could affect the risk of arm lymphedema in comparison to targeting only the whole breast, given the documented location of arm lymphatic drainage in the axilla [23,24].

This suggestion of limited-field RNI using IMRT may also be applied to cases where neoadjuvant chemotherapy is utilized. Historically, the pre-neoadjuvant chemotherapy stage has been used to determine the need for RNI. However, post hoc analyses of the NSABP B-18 and B-27 trials have demonstrated that locoregional recurrence as a first event was rare in patients with down-staged pathologically negative nodes (even in those with residual disease in the breast) who did not receive RNI [25]. There is a growing interest in avoiding RNI in patients with initial cN1 disease who convert to ypN0, thus minimizing added toxicity with more extensive fields. To address this question, the NSABP B-51 trial was designed for patients with biopsy-confirmed nodal metastasis who convert to ypN0 following neoadjuvant chemotherapy. In this clinical trial, breast conserving surgery patients are randomized to WBI with or without RNI [26]. The results of NSABP B-51 may take years to mature, and until then, there is currently no consensus among clinicians on appropriate RT volumes for ypN0 patients who undergo sentinel lymph node biopsy only [25]. In the meantime, including axillary levels I-II in the target may be a reasonable option when using hypofractionated IMRT.

## 5. Conclusions

In conclusion, this investigation indicates that tomotherapy—specifically, the TomoDirect approach—administers an incidental axillary dose at a low level that is comparable to other IMRT studies. Relying on the unpredictable incidental dose for disease control, rather than on the prescribed dose, can introduce considerable uncertainties when estimating the efficacy of RT. Clinical trials are required to determine the appropriate level of axillary dose for each risk group among patients with early breast cancer. These results could enable IMRT planning with risk-adjusted axilla coverage, which can help to attain regional control and reduce critical organ toxicity.

## Figures and Tables

**Table 1:** Patient characteristics (*n* = 30).

Characteristics		N (%)
Age, median (range)		51 (40–66)
Laterality	Left	18 (60.0)
	Right	12 (40.0)
pT stage *	T1a	2 (6.7)
	T1b	10 (33.3)
	T1c	10 (33.3)
	T2	8 (26.7)
pN stage *	N0	28 (93.3)
	Nmi	2 (6.7)
Pathology	Invasive ductal carcinoma	26 (86.6)
	Invasive lobular carcinoma	2 (6.7)
	Invasive carcinoma, no special type	2 (6.7)

* American Joint Committee on Cancer (AJCC) Cancer Staging Manual, 8th ed.

**Table 2:** Dose-volume data of axillary lymph nodes.

Axilla Levels	Dmean, % (Gy ± SD)	V95%[%]	V90%[%]	V80%[%]	V50%[%]
Level I	35.5 (15.5 ± 4.8)	4.7 ± 3.9	7.7 ± 5.8	14.0 ± 8.6	35.3 ± 14.4
Level II	35.1 (14.9 ± 4.2)	4.8 ± 3.7	9.2 ± 5.1	18.0 ± 7.8	36.1 ± 11.9
Level III	3.5 (1.5 ± 1.6)	0 ± 0	0 ± 0	0 ± 0	0.7 ± 2.3

Dmean = mean dose; SD = standard deviation; Vx%[%] = volume[%] receiving x% of prescription dose.

**Table 3:** Results of representative studies.

RT Techniques	Axilla Levels	Dmean, % (Gy)	V95%[%]	V90%[%]	V80%[%]
HT [9]	Level I	86	79	ND	ND
	Level II	71	51	ND	ND
	Level III	73	49	ND	ND
ST [9]	Level I	66	51	ND	ND
	Level II	44	26	ND	ND
	Level III	31	15	ND	ND
s-IMRT [10]	Level I	55.4 (27.7)	16.9	22.1	31.3
	Level II	21.2 (10.6)	1.7	2.7	5.7
	Level III	5 (2.5)	0	0	0.1
VMAT * [11]	Level I	26.66	2.6	6	ND
	Level II	17.83	2.6	2.4	ND
	Level III	5.96	ND	ND	ND

RT = radiation therapy, Dmean = mean dose; HT = high tangents; ST = standard tangents; s-IMRT = static intensity-modulated radiation therapy; VMAT = volumetric modulated arc therapy; Vx%[%] = volume[%] receiving x% of prescription dose; ND = no data. * Median values.

## Data Availability

Data are available upon reasonable request.
